# Cytotoxic and pro-apoptotic action of MjTX-I, a phospholipase A2 isolated from *Bothrops moojeni* snake venom, towards leukemic cells

**DOI:** 10.1186/s40409-018-0180-9

**Published:** 2018-12-20

**Authors:** Rogério Bodini Benati, Tássia Rafaela Costa, Maira da Costa Cacemiro, Suely Vilela Sampaio, Fabíola Attié de Castro, Sandra Mara Burin

**Affiliations:** Departamento de Análises Clínicas, Toxicológicas e Bromatológicas. Faculdade de Ciências Farmacêuticas de Ribeirão Preto, Universidade de São Paulo, Ribeirão Preto, SP Brazil

**Keywords:** Chronic myeloid leukemia, Bcr-Abl, Phospholipase A_2_, MjTX-I, *Bothrops moojeni*, Apoptosis, Cytotoxicity

## Abstract

**Background:**

Chronic myeloid leukemia (CML) is a *BCR-ABL1*^*+*^ myeloproliferative neoplasm marked by increased myeloproliferation and presence of leukemic cells resistant to apoptosis. The current first-line therapy for CML is administration of the tyrosine kinase inhibitors imatinib mesylate, dasatinib or nilotinib. Although effective to treat CML, some patients have become resistant to this therapy, leading to disease progression and death. Thus, the discovery of new compounds to improve CML therapy is still challenging. Here we addressed whether MjTX-I, a phospholipase A_2_ isolated from *Bothrops moojeni* snake venom, affects the viability of imatinib mesylate-resistant Bcr-Abl^+^ cell lines.

**Methods:**

We examined the cytotoxic and pro-apoptotic effect of MjTX-I in K562-S and K562-R Bcr-Abl^+^ cells and in the non-tumor HEK-293 cell line and peripheral blood mononuclear cells, using the 3-(4,5-dimethylthiazol-2-yl)-2,5-diphenyltetrazolium bromide and the hypotonic fluorescent solution methods, associated with detection of caspases 3, 8, and 9 activation and poly (ADP-ribose) polymerase (PARP) cleavage. We also analyzed the MjTX-I potential to modulate the expression of apoptosis-related genes in K562-S and K562-R cells.

**Results:**

MjTX-I decreased the viability of K562-S and K562-R cells by 60 to 65%, without affecting the viability of the non-tumor cells, i.e. it exerted selective cytotoxicity towards Bcr-Abl^+^ cell lines. In leukemic cell lines, the toxin induced apoptosis, activated caspases 3, 8, and 9, cleaved PARP, downregulated expression of the anti-apoptotic gene *BCL-2*, and upregulated expression of the pro-apoptotic gene *BAD*.

**Conclusion:**

The antitumor effect of MjTX-I is associated with its potential to induce apoptosis and cytotoxicity in Bcr-Abl positive cell lines sensitive and resistant to imatinib mesylate, indicating that MjTX-I is a promising candidate drug to upgrade the CML therapy.

## Background

Chronic myeloid leukemia (CML) is a *BCR-ABL1*^*+*^ myeloproliferative neoplasm [1], characterized by increased myeloproliferation rate and presence of apoptosis-resistant leukemic cells [2, 3]. The current CML treatment relies on administration of the tyrosine kinase inhibitors imatinib mesylate (IM), dasatinib or nilotinib as first-line therapy. IM has been efficient to manage CML, but some patients have developed resistance to this therapy; when the therapeutic intervention fails, CML patients progress to the blast phase, which is almost always fatal [2, 4–6]. The main causes of resistance are related to either mutations at the Bcr-Abl catalytic site, such as the T315I, or to *BCR-ABL1* duplication or overexpression [7, 8]. Despite all the advances and successes in CML therapy, it remains a challenge to find an efficient treatment to CML patients who are resistant to tyrosine kinase inhibitors.

The antitumor effect of snake venoms has been explored since the last century [9–11]. Snake venoms hold many bioactive proteins, among which the phospholipase A_2_ (PLA_2_) isoforms, also called myotoxins, are one of the most abundant components [12, 13]. PLA_2_ not only exerts toxic and digestive effects, but also exhibits pharmacological and cytotoxic activity [14–16]. Studies have reported the cytotoxic and pro-apoptotic effects of a variety of PLA_2_ isolated from snake venoms in different tumor cell lines such as HL-60 (human promyelocytic leukemia), HepG2 (human hepatoma), PC12 (adrenal phaeochromocytoma), B16F10 (melanoma), Jurkat (acute T cell leukemia), SKBR-3 (human breast cancer), and Ehrlich ascites tumor [17–22].

The PLA_2_ isoforms are divided into two categories: neurotoxic (family Elapidae – genus *micrurus*) and non-neurotoxic (family Viperidae – genera *Crotalus* and *Bothrops*) [23]. The isoforms isolated from snakes belonging to the genus *Bothrops* are the main venom components that account for cell damage mediated by hydrolysis of membrane phospholipids [24]. The MjTX-I isolated from *Bothrops moojeni* snake venom (*B. moojeni* myotoxin I) is genotoxic to human lymphocyte DNA. BthTX-I and BthTX-II isolated from *Bothrops jararacussu* snake venom also damage lymphocyte DNA [25]. The mechanisms by which toxins isolated from snake venoms cause genotoxicity have not been elucidated yet, but they are probably related to the toxin-mediated free radical production [25–27].

Considering the need to search for new molecules to treat CML, and the knowledge that MjTX-I is cytotoxic, here we examined whether this myotoxin exerts antitumor effect against the Bcr-Abl^+^ cell lines sensitive (K562-S) or resistant (K562-R) to imatinib mesylate, a drug used as first-line treatment for CML.

## Material and methods

### Cell lines

This study used the cell lines K562-S (IM-sensitive Bcr-Abl^+^ cells) and K562-R (IM-resistant Bcr-Abl^+^ cells), isolated from CML patients in blast phase who were sensitive or resistant to IM treatment, respectively. The cell lines were kindly provided by Dr. JPGAM. HEK-293 cells, derived from embryonic epithelial cells of human kidney, were acquired from the Rio de Janeiro Cell Bank (BCRJ: 0009) and kindly provided by Professor AML.

K562-S and K562-R cells were cultured in complete RPMI (*Roswell Park Memorial Institute*) 1640 medium, while HEK-293 cells were cultured in complete DMEM (*Dulbecco’s Modified Eagle Medium*). Both culture media were supplemented with 10% fetal bovine serum and 1% penicillin/streptomycin, and the three cell lines were incubated under an atmosphere of 5% CO_2_ and 95% air, at 37 °C.

### Isolation and purification of MjTX-I

*Bothrops moojeni* snake venom was donated by the Center for the Study of Venoms and Venomous Animals (CEVAP) from São Paulo State University (UNESP), Botucatu, São Paulo, Brazil, and stored at − 20 °C. The MjTX-I (*B. moojeni* myotoxin I) was purified from *Botrops moojeni* crude venom through anion-exchange chromatography on CM-Sepharose (Pharmacia) adapted from Lomonte et al. [28]. The eluted toxin homogeneity was analyzed by SDS–PAGE and reversed-phase chromatography.

### Isolation of peripheral blood mononuclear cells (PBMC)

Peripheral blood was collected into vacuum tubes containing anticoagulant, from three healthy individuals aged between 30 and 40 years after their consent. The human peripheral blood mononuclear cells (PBMC) were isolated by the Ficoll-Hypaque density gradient centrifugation method, using Histopaque-1077 (Sigma Diagnostics, Inc., MO, USA). After centrifugation (500 x g for 30 min), the PBMC-rich layer was collected and suspended in 1 mL of complete RPMI. Cells were diluted in Turk’s dye and counted in the Neubauer’s chamber. Next, the cells were plated, treated with MjTX-I, and incubated under an atmosphere of 5% CO_2_, at 37 °C.

### Cytotoxicity assay

Cell viability was assessed using the 3-(4,5-dimethylthiazol-2-yl)-2,5-diphenyltetrazolium bromide (MTT) method described by Mosmann [29], with modifications. The tumor cell lines K562-S and K562-R and the non-tumor cells HEK-293 and PBMC (2 × 10^4^ cells) were treated for 24 h with MjTX-I at different concentrations (3.15, 6.25, 12.5, 25, 50, 75, 100, 150, 200, 300, and 400 μg/mL). Untreated cells were used as negative control, and cells treated with 1% Triton X-100 (Bio-Rad, Hercules, CA, USA) were used as positive control. Next, 20 μL of MTT (5 mg/mL) were added to each well, and the microplate was incubated for 4 h, at 37 °C. The supernatants were discarded, and the formazan crystals were dissolved with 200 μL of dimethyl sulfoxide. After 30 min of incubation at room temperature, the absorbance was recorded at 570 nm. The percentage of cell viability was used to calculate the toxin concentration capable of killing 50% of the cells (IC_50_). The IC50 was calculated by using the Calcusyn 2.1 software.

### Apoptosis analyses

#### Flow cytometric quantification of apoptosis

The toxin potential to sensitize cells to and induce apoptosis was quantified using the hypotonic fluorescent solution (HFS) method reported by Riccardi and Nicoletti [30]. K562-S and K562-R cells (1 × 10^5^ cells) were treated for 24 h with MjTX-I at 6.25, 12.5, 50, and 100 μg/mL, as well as at the concentrations corresponding to the IC_50_ values for these cell lines: 257 and 191 μg/mL, respectively. Untreated cells were used as the negative control, and 25 μM Etoposide (VP-16) was used as the cell death control. Next, the cells were recovered, transferred to flow cytometry tubes, and suspended in 400 μL of HFS solution (50 μg/mL propidium iodide, 0.1% sodium citrate, and 0.1% Triton X-100). After a 15-min incubation in the dark, at 4 °C, cells were analyzed in the FACSCanto flow cytometer (BD, Sunnyvale, CA, USA), with the aid of the FACSDiva software (BD, San Diego, CA, USA). Five thousand events were acquired and the cell population was analyzed to determine the percentage of hypodiploid nuclei (apoptotic nuclei).

#### Western blotting detection of caspase activation and poly (ADP-ribose) polymerase (PARP) cleavage

K562-S and K562-R cells (1 × 10^6^ cells) were treated for 24 h with MjTX-I (6.25, 12.5, 50, 100, 257, and 191 μg/mL), VP-16 25 μM (positive control), or culture media (negative control). Afterward, the cells were collected and suspended in the western blotting lysis buffer (20 mM Tris–HCl pH 7.4, 150 mM NaCl, 1 mM EDTA, and phosphatase and protease inhibitors). Total protein concentration in the samples was determined using the BCA protein assay reagent, according to the manufacturer’s instructions (Thermo Fischer Scientific, Waltham, MA, USA). Equal amounts of protein were analyzed by 15% SDS-PAGE (sodium dodecyl sulfate polyacrylamide gel electrophoresis), where they were separated according to their molecular weight. Next, proteins were transferred to polyvinylidene difluoride (PVDF) membranes (Amersham, GE Healthcare Life Science, Pittsburgh, PA, USA). To detect the proteins, the membranes were first blocked for 2 h with 5% non-fat dry milk prepared in Tris-buffered saline with Tween-20 (20 mM Tris, 137 mM NaCl, 0.01% Tween-20). The PVDF membranes were incubated overnight, at 4 °C, with the following primary antibodies acquired from Cell Signaling Technology (Danvers, MA, USA): anti-caspase 3 (code 96625), anti-caspase 8 (code 9746), anti-caspase 9 (code 9502), anti-PARP (code 9541), and anti-β-tubulin (code 2146). Then, the PVDF membranes were incubated with the appropriate secondary antibodies and the expression of target proteins were detected using ECL (Amersham, GE Healthcare Life Science, Pittsburgh, PA, USA). The protein tubulin was used as an internal standard to normalize protein load among samples.

### Expression of apoptosis-related genes

#### Total RNA extraction

Total RNA from K562-S and K562-R cells (1 × 10^6^ cells) treated with MjTX-I at low concentrations (6.25 and 12.5 μg/mL) was extracted using the Trizol® method, following the manufacturer’s instructions (Invitrogen Life Technologies®, Carlsbad, USA). Untreated cells were used as the negative control. RNA concentration of all samples was determined by the absorbance ratio determined at 260 nm and 280 nm (A260/A280), using the NanoVue spectrophotometer (GE Healthcare Life Sciences, Pittsburgh, PA, USA).

#### Complementary DNA (cDNA) synthesis and real-time polymerase chain reaction (PCR)

Total RNA (1 μg) was reverse transcribed to cDNA synthesis using the High Capacity cDNA reverse transcription® assay kit (Applied Biosystems®, Foster City, USA), according to the manufacturer’s instructions. cDNA (diluted 1:4) was used in the real time PCR assay to analyze expression of apoptosis-related genes: *BAD, BAX* (pro-apoptotic members from Bcl-2 family), *BCL-2*, *BCL-X*_*L*_ (anti-apoptotic members from Bcl-2 family), and the *c-FLIP* (anti-apoptotic gene from apoptosis extrinsic pathway). Gene expression was quantified by real time PCR (three experiments assayed in duplicate) using the SYBR Green PCR Master Mix Kit (Applied Biosystems, Carlsbad, CA, USA) and the StepOnePlus™ equipment (Applied Biosystems). The results were normalized by the geometric mean of the β-actin and the B2M housekeeping genes expression and represented by 2-ΔΔCt. The sequences of the specific oligonucleotides (Invitrogen Life Technologies) used for quantification of gene expression are listed in Table 1.

**Table 1:** Oligonucleotide sequences used for quantification of gene expression

*Target Gene*	Oligonucleotide Sequence (5′ to 3′)
*BAD*	F: CCG AGT GAG CAG GAA GAC TC R: GGT AGG AGC TGT GGC GAC T
*BAX*	F: CCC TTT TGC TTC AGG GTT TC R: TCT TCT TCC AGA TGG TGA GTG
*BCL-2*	F: ACG AGT GGG ATG CGG GAG ATG TG R: GCG GTA GCG GCG GGA GAA GTC
*BCL-X*_*L*_	F: CTG AAT CGG AGA TGG AGA CC R: TGG GAT GTC AGG TCA CTG AA
*c-FLIP*	F: GCC GAG GCA AGA TAA GCA R: GCC CAG GGA AGT GAA GGT
*β-ACTIN*	F: GCC CTG AGG CAC TCT TCC A R: CCA GGG CAG TGA TCT CCT TCT
*B2M*	F: CCA GCG TAC TCCC AAA GAT TCA R: TGG ATG AAA CCC AGA CAC ATA G

*F* Forward Primer, *R* Reverse Primer

### Statistical data analysis

Experimental data were compared using One-way Analysis of Variance (ANOVA) followed by the Tukey’s *post-hoc* test, with the aid of the GraphPad Prism software, version 5.0 (GraphPad Software, San Diego, California, USA). *p* < 0.05 were considered as statistically significant.

## Results

### MjTX-I is cytotoxic towards leukemic cells but not towards non-tumor cells

**Fig. 1:**
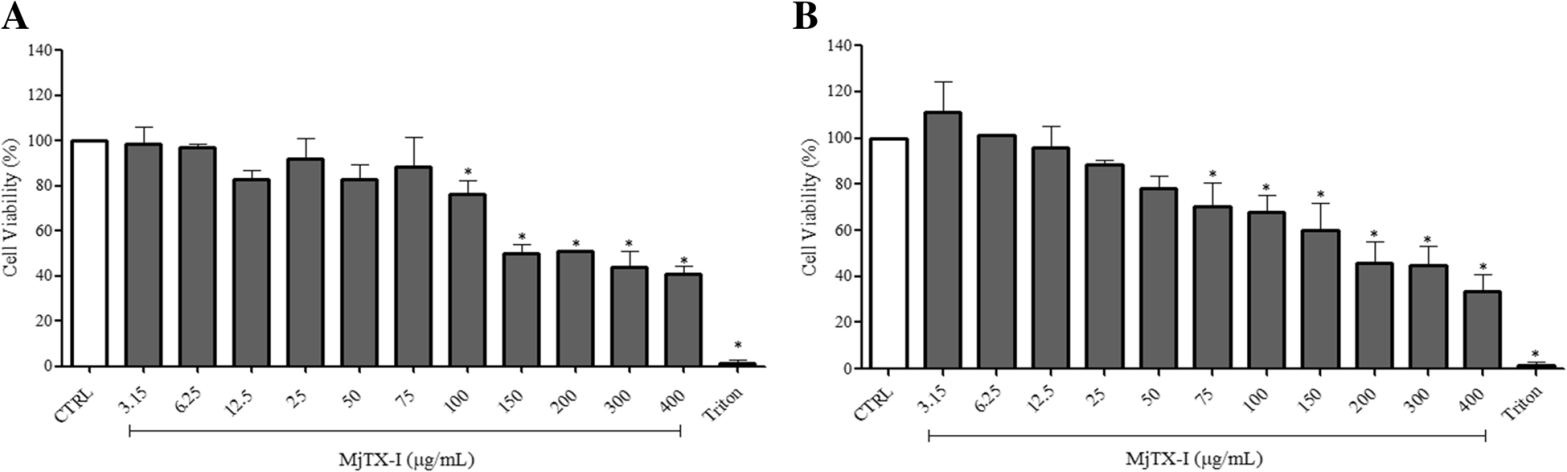
Cytotoxicity of MjTX-I towards (**a**) K562-S (**b**) K562-R tumor cell lines. Results are expressed as the mean percentage of cell viability ± standard deviation of three independent experiments assayed in triplicate. CTRL: untreated cells (negative control). *p < 0.05 vs. CTRL (One-way ANOVA followed by the Tukey’s *post-hoc* test)

**Fig. 2:**
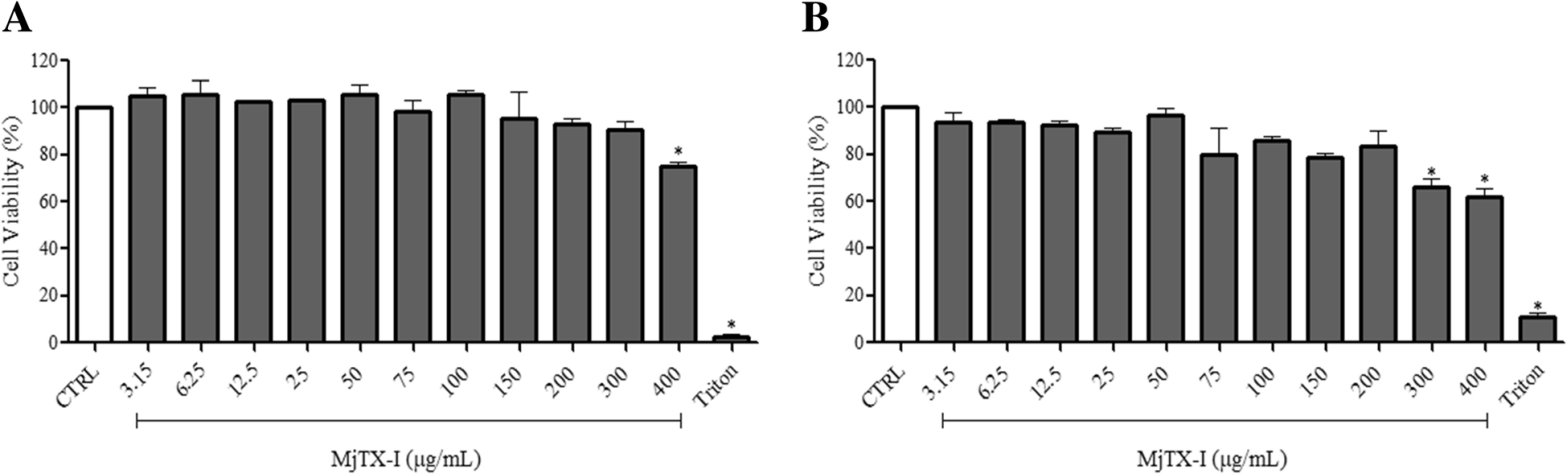
Cytotoxicity of MjTX-I towards (**a**) HEK-293 and (**b**) PBMC non-tumor cells. Results are expressed as the mean percentage of cell viability ± standard deviation of three independent experiments assayed in triplicate. CTRL: untreated cells (negative control). *p < 0.05 vs. CTRL (One-way ANOVA followed by the Tukey’s *post-hoc* test)

We examined the cytotoxicity of MjTX-I towards the tumor cell lines K562-S and K562-R, and towards the non-tumor cells HEK-293 and PBMC, after a 24-h treatment with toxin concentrations ranging from 3.15 to 400 μg/mL. The percentage of cell viability of K562-S and K562-R cells treated with the toxin at concentrations higher than 100 μg/mL and 75 μg/mL, respectively, decreased significantly (*p* < 0.05), and reached 40 and 35% after treatment with 400 μg/mL of the toxin, respectively. The IC_50_ values for K562-S and K562-R cells were 257 μg/mL and 191 μg/mL, respectively (Fig. 1a and b). In non-tumor cells, MjTX-I reduced cell viability of HEK-293 cells by 25% at the highest concentration tested (400 μg/mL), and reduced cell viability of PBMC by 34–38% at the two highest concentrations tested (300 and 400 μg/mL) (Fig. 2a and b).

### MjTX-I induces cell death in leukemic cell lines

**Fig. 3:**
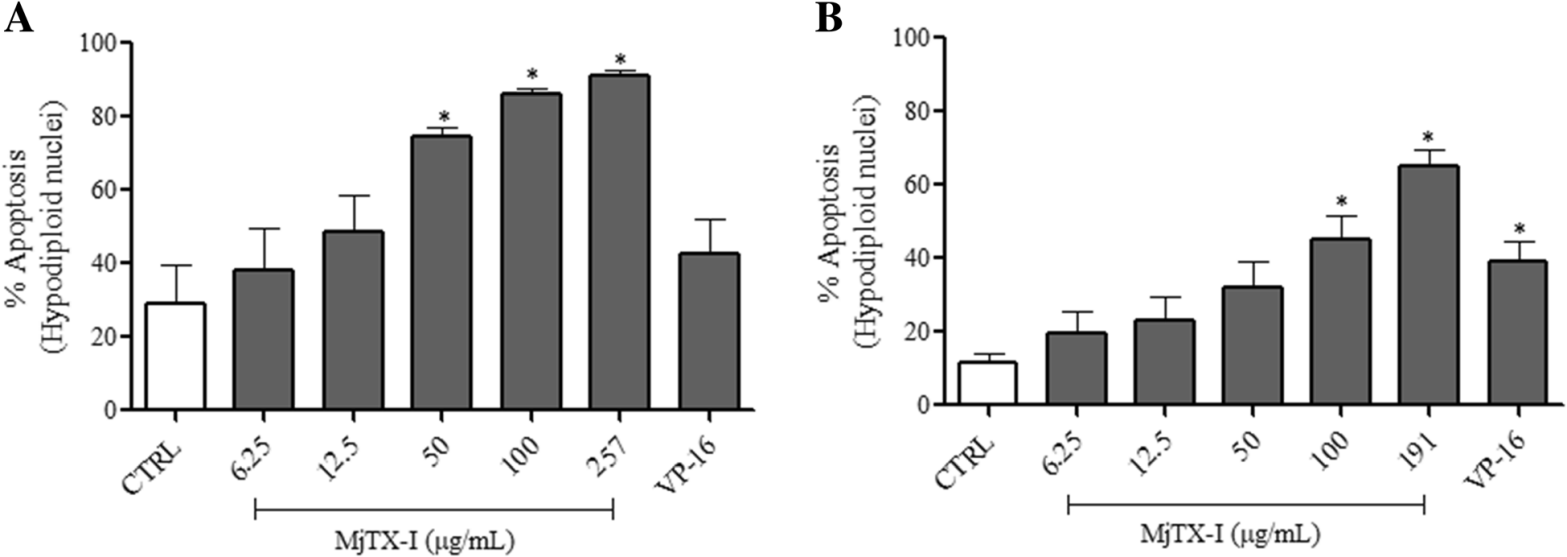
Quantification of MjTX-I-induced apoptosis in (**a**) K562-S and (**b**) K562-R cells, assessed by the hypotonic fluorescent solution (HFS) method. Results are expressed as mean percentage of cells with hypodiploid nuclei ± standard deviation of three independent experiments. CTRL: untreated cells (negative control). VP-16: etoposide (positive control). *p < 0.05 vs. CTRL (One-way ANOVA followed by the Tukey’s *post-hoc* test)

After analyzing the cytotoxicity of MjTX-I, we examined whether it sensitized Bcr-Abl^+^ cell lines to apoptosis. Cell death was assessed by quantifying formation of hypodiploid nuclei (apoptotic nuclei). Compared with the control, the percentage of hypodiploid nuclei increased by 45.5–62% in K562-S cells treated with 50–257 μg/mL of the toxin (*p* < 0.05; Fig. 3a), and by 34 and 54% in K562-R cells treated with 100 and 191 μg/mL of the toxin, respectively (p < 0.05; Fig. 3b). MjTX-I at low concentrations promoted a weak but not significant increase in the percentage of hypodiploid nuclei (10–20%) (Fig. 3a and b).

### MjTX-I induces caspases activation in leukemic cell lines

We examined the activation of caspases 3, 8, and 9 and PARP cleavage in K562-S and K562-R cells to confirm apoptosis induction, as wells as to determine which apoptosis pathway was activated – intrinsic or extrinsic – in these cells. MjTX-I at 100 and 257 μg/mL induced high levels of cell death, which thereby impaired preparation of cell lysate and protein quantification.

**Fig. 4:**
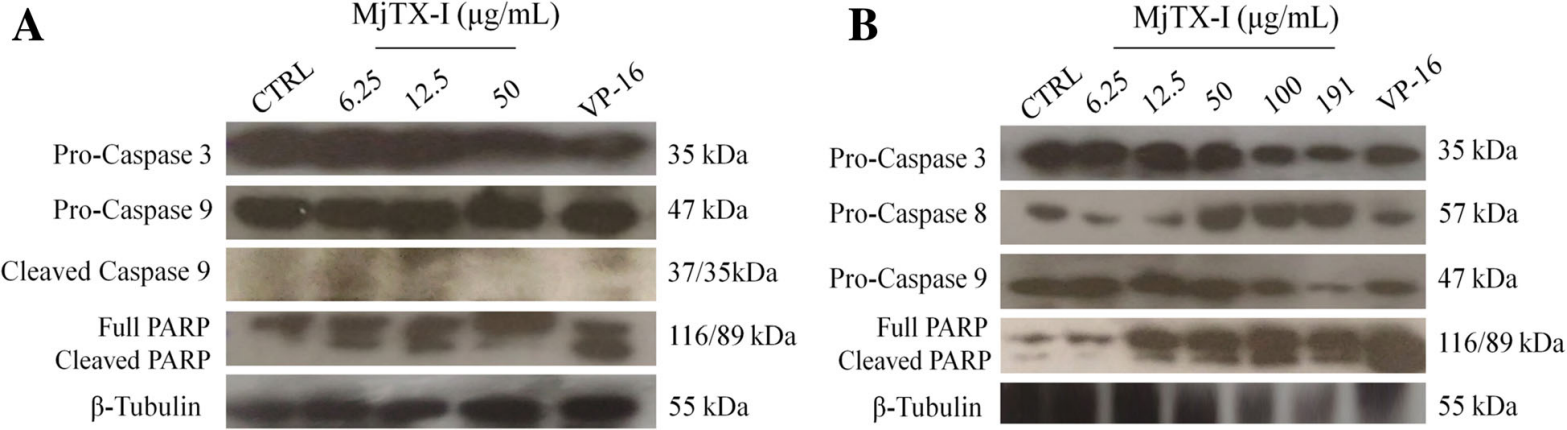
Western blotting analysis of protein expression in (**a**) K562-S and (**b**) K562-R cells treated with MjTX-I. Detection of expression of caspases 3, 8, and 9 and PARP after a 24-h treatment with MjTX-I. CTRL: untreated cells (negative control). VP16: etoposide (positive control)

In K562-S cells, MjTX-I at 50 μg/mL lowered the levels of pro-caspase 3 expression, while the toxin at 6.25 and 12.5 μg/mL increased the levels of caspase-9 expression and PARP cleavage (Fig. 4a). In K562-R cells, the toxin lowered the levels of pro-caspase 3 and pro-caspase 9 expression at 100 and 191 μg/mL, lowered the levels of pro-caspase 8 expression at 6.25 and 12.5 μg/mL, and increased the levels of cleaved PARP at concentrations greater than 12.5 μg/mL (Fig. 4b). Treatment with VP-16 lowered the levels of pro-caspase 3 and increased the levels of cleaved PARP in K562-S and K562-R cells (Fig. 4a and b); in the former cell line, it also augmented the levels of caspase 9 expression (Fig. 4a).

The disappearance of specific bands of caspases proforms and the appearance of bands of the cleaved forms indicated caspases activation. These findings suggest that MjTX-I induced apoptosis by activating the intrinsic and extrinsic pathways.

### MjTX-I modulates expression of apoptosis-related genes

**Fig. 5:**
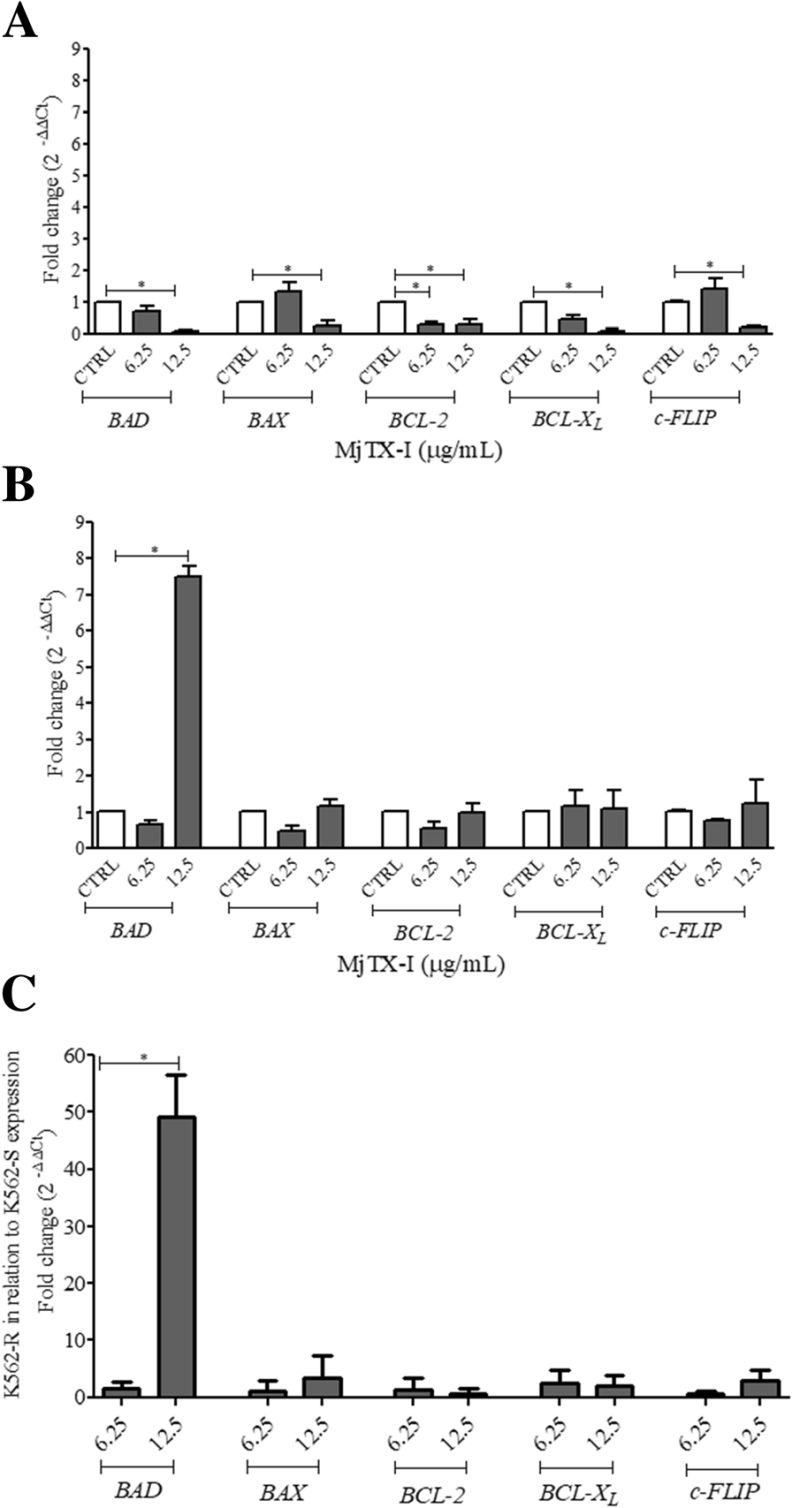
Expression of apoptosis-related genes in K562-S and K562-R cells treated with MjTX-I. Expression of *BAD*, *BAX*, *BCL-2*, *BCL-X*_*L*_, and *c-FLIP* genes was analyzed by real-time PCR after a 24-h treatment with MjTX-I. (**a**) K562-S cells. (**b**) K562-R cells. (**c**) Gene expression ratio between K562-R and K562-S cells (fold change). Results are expressed as mean ± standard deviation of three independent experiments. CTRL: untreated cells (control). *p < 0.05 vs. CTRL (One-way ANOVA followed by the Tukey’s *post-hoc* test)

After confirming that MjTX-I was capable of inducing cell death in leukemic cells, we selected sub lethal concentrations that sensitized cells to apoptosis – 6.25 and 12.5 μg/mL – and examined whether they modulated the expression of pro- and anti-apoptotic genes in K562-S and K562-R cells. In K562-S cells, MjTX-I lowered the expression level of the anti-apoptotic gene *BCL-2* (fold change = 0.32) at 6.25 μg/mL, and lowered the expression levels of all the genes analyzed herein at 12.5 μg/mL: *BAD* (fold change = 0.11), *BAX* (fold change = 0.27), *BCL-2* (fold change = 0.31), *BCL-X*_*L*_ (fold change = 0.11), and *c-FLIP* (fold change = 0.21) (Fig. 5a). MjTX-I at 12.5 μg/mL also increased the expression level of the pro-apoptotic gene *BAD* (fold change = 7.5) in K562-R cells (Fig. 5b), which was 49% greater than that detected in K562-S cells (Fig. 5c).

## Discussion

Toxins isolated from snake venom, such as PLA_2_, have been prominent as potent antitumor agents in the last years. Scientists have investigated this enzyme class as a promising tool to develop new candidate drugs to treat cancer [13, 18, 31]. The present study examined the cytotoxic and pro-apoptotic effect of the myotoxin MjTX-I isolated from *Bothrops moojeni* snake venom, with the purpose of better understanding its mechanism of action and describing a molecule that could help to treat CML.

We found that MjTX-I was cytotoxic towards the leukemic cell lines K562-S and K562-R and reduced their viability by 60 and 65%, respectively. The literature reports the antitumor potential of PLA_2_ isolated from snake venom towards other tumor cell lines. For instance, BthTX-I at 10–100 μg/mL reduces cell viability of the tumor cell lines HL-60, PC12, and B16F10 by 40–60% [18]; two PLA_2_ isolated from *Bothrops brazili*, named as MTX-I and MTX-II, at 100 μg/mL are cytotoxic towards Jurkat cells [20]; BthA-IPLA_2_, the acidic PLA_2_ isolated from *Bothrops jararacussu*, at 100 μg/mL is cytotoxic towards Jurkat, SKBR-3, and Ehrlich ascites tumor cells [22].

The cytotoxic mechanisms of PLA_2_ are poorly understood. This enzyme class directly acts on the membrane phospholipid metabolism and interferes with lipid biosynthesis in a variety of cell lines, including tumor cell lines [31]. The myotoxin C-terminal region can disrupt the membrane hydrophilic matrix and cause the opening pores and the toxin entry into the intracellular environment [32]. In other words, one hypothesis is that the interaction between the toxin C-terminal region and cell membranes mediate the cytotoxicity of myotoxins [20, 21, 33]. Another possibility is that PLA_2_ triggers the production of reactive oxygen species and induces oxidative stress, which are associated with the cytotoxic effects [25–27].

Considering that toxins isolated from snake venoms are potential candidates for the development of new drugs, and that their administration may pose risk to human health, their cytotoxic effects should also be assessed in non-tumor cells. Here we demonstrated that the non-tumor cells HEK-293 and PBMC were resistant to the cytotoxic effect of MjTX-I, since toxin concentrations as high as 300 and 400 μg/mL decreased cell viability by no more than 38%.

There are few reports on the action of PLA_2_ on non-tumor cells. In line with our findings, there are evidences that PLA_2_ is more strongly cytotoxic towards tumor cells than towards non-tumor cells. The non-tumor cell lines HEK-293 and C2C12 (mouse skeletal muscle cell) were resistant to the cytotoxic effects of AtxA, a PLA_2_ isolated from *Vipera ammodytes ammodytes* that is strongly cytotoxic towards the tumor cell line NSC34 (Neuroblastoma) [34]. Some studies on the cytotoxicity, genotoxicity, and mutagenicity of some PLA_2_ in human lymphocytes have revealed that CB PLA_2_ and CA-crotapotin – two PLA_2_ isolated from *Crotalus durissus terrificus* – are not cytotoxic and cause reversible DNA damage [27]; the PLA_2_ BthTX-I, BthTX-II, and MjTX-I are not cytotoxic; and MjTX-I causes weaker DNA damage than the other PLA_2_. In this sense, the findings of the present study may have relevant clinical implications because one of the requirements of cancer therapy is the selective toxicity to tumor cells, i.e. low toxicity to non-tumor cells.

Next, we examined the pro-apoptotic effect of MjTX-I to address whether it was capable of sensitizing cells to and/or inducing apoptosis. Quantification of apoptosis through the HFS method revealed that MjTX-I increased the formation of hypodiploid nuclei (apoptotic) in K562-S and K562-R cells, being the former more sensitive to the effect of the toxin. VP-16 also induced the formation of hypodiploid nuclei in both cell lines, but less strongly than the MjTX-I concentration that exerted significant effects; these findings confirm the resistance of both tumor cell lines to conventional chemotherapeutic agents.

As apoptosis is a dynamic process where the cellular events occur within a short time-window, different methods should be used to confirm this process. For this reason, here we analyzed the expression of the apoptotic proteins caspases 3, 8, and 9 and PARP by western blotting as well as the expression of pro- and anti-apoptotic genes by real-time PCR. Activation of caspases 3 and 9 followed by PARP cleavage in K562-R and K562-S cells indicates triggering of the intrinsic apoptosis pathway; in addition, activation of caspase 8 in K562-R cells suggests triggering of the extrinsic apoptosis pathway.

In line with the findings reported in the previous paragraph, we found that MjTX-I modulated the expression of pro- and anti-apoptotic genes. MjTX-I at both concentrations tested modulated gene expression in K562-S cells. This cell line was more sensitive to the toxin effect at 12.5 μg/mL, which downregulated the expression of not only the anti-apoptotic genes *BCL-2*, *BCL-XL*, and *c-FLIP*, but also of the pro-apoptotic genes *BAD* and *BAX*. The toxin at 6.25 μg/mL did not interfere in the expression of the pro-apoptotic genes but downregulated *BCL-2* expression. MjTX-I did not modulate expression of the anti-apoptotic genes but increased *BAD* expression by 7.5-fold in K562-R cells.

K562-S and K562-R are the particular K562 cell sublines that are less or more resistant to chemotherapy drugs in vitro, respectively [35, 36]. In K562-S and K562-R cells that are sensitive and resistant to imatinib mesylate, respectively, the present study demonstrated that treatment with similar MjTX-I concentrations differently modulated gene expression, and evidenced that the toxin increased expression of a pro-apoptotic gene in resistant cells more effectively than in sensitive cells. The K562-S and K562-R cells response to MjTX-I treatment differed probably due to karyotype variability [37] and the role that some membrane proteins play in K562-R cell resistance [35].

Tyrosine kinase activity of Bcr-Abl in CML is associated with apoptosis inhibition by increasing the expression of the anti-apoptotic proteins Bcl-2 and Bcl-X_L_ [38, 39], which play critical roles in the mitochondrial apoptosis pathway [40]. It is worth to note that Bcr-Abl positive cell lines are more resistant to cell death induced by different apoptosis inducers [41].

The anti-apoptotic protein Bcl-2 acts by suppressing the pro-apoptotic protein complex Bax/Bak; however, Bcl-2 inhibition activates this complex and induces apoptosis [42, 43]. The pro-apoptotic protein Bad acts on the cytosol, more specifically it directly acts on the mitochondria and helps to inhibit Bcl-2 and Bcl-X_L_; it thereby activates the intrinsic apoptosis pathway [44, 45]. In this sense, the fact that MjTX-I activated caspase 3 through the intrinsic and extrinsic apoptosis pathways, downregulated *BCL-2* expression, and upregulated *BAD* expression indicate that this toxin is a promising molecule for the adjuvant treatment of CML.

Other studies have demonstrated the pro-apoptotic effect of PLA_2_. BthTX-I at 25, 50, and 100 μg/mL elicits apoptosis in the tumor cell lines PC-12, B16F10, HL-60, and HepG2 [18]. The phospholipase MT-II (homologue of PLA_2_ from *Bothrops asper*) induces apoptosis and cell proliferation, depending on the toxin concentration tested, as assessed by the TUNEL method [46]. AtxA at 100 nmol/L exerts cytotoxicity associated with apoptosis induction in the tumor cell line NSC34 [34].

The loss of mitochondrial membrane potential and caspase 3 activation confirmed our findings. Corroborating our data, CMS-9 (a PLA_2_ isolated from *Naja nigricollis* venom) at 0.1 μM induces apoptosis in K562 cells. Mitochondrial depolarization and activation of caspases 3 and 9 confirmed the pro-apoptotic action of this toxin. CMS-9 also modulates expression of pro- and anti-apoptotic proteins: it lowers Bcl-2 expression and increases expression and mitochondrial translocation of Bax [47].

The mechanisms by which PLA_2_ induces apoptosis and exerts cytotoxicity in tumor cell lines are not completely understood. Some authors propose that PLA_2_-induced apoptosis is related to the cytotoxic effects of these enzymes [48], while others believe that PLA_2_ accelerates phospholipid turnover and influences on the membrane alterations that occur during the apoptotic process [20, 49]. Another hypothesis is that the pro-apoptotic action of PLA_2_ is associated with oxidative stress caused by the release of reactive oxygen species and increase of intracellular Ca^2+^ concentration in the mitochondrial matrix region due to formation of membrane permeability transition pores [25, 47, 50].

Together, the results reported herein stress the apoptosis-inducing capacity of PLA_2_ and contribute to better understand the mechanisms by which this toxin class act. Therefore, MjTX-I can be considered as an enzyme with promising therapeutic applications.

## Conclusion

MjTX-I exerts selective cytotoxicity against leukemic cell lines, with low toxicity towards non-tumor cells, and induces apoptosis accompanied by caspases activation and downregulation of *BCL-2* and upregulation of *BAD* expression. Our findings add important knowledge to the mechanisms underlying the action of snake venom phospholipases, as well as help to improve the CML therapy.

## Abbreviations

AtxA: Phospholipase isolated from *Vipera ammodytes ammodytes*
BthTX-I and BthTX-II: Phospholipases isolated from *Bothrops jararacussu*
cDNA: Complementary DNA
CML: Chronic myeloid leukemia
CMS-9: Phospholipase isolated from *Naja nigricollis*
HFS: Hypotonic fluorescent solution
IC_50_: Toxin concentration that kills 50% of the cell lines
IM: Imatinib mesylate
MjTX-I: Myotoxin PLA_2_-Lys49 isolated from *Bothrops moojeni*
MTT: (3-(4,5-dimethylthiazol-2-yl)-2,5-diphenyltetrazolium bromide
PARP: Poly-(ADP-ribose)-polymerase
PBMC: Peripheral blood mononuclear cells
PCR: Polymerase chain reaction
PLA_2_: Phospholipase A_2_
PVDF: Polyvinylidene difluoride
VP-16: Etoposide
